# Quantum coding with finite resources

**DOI:** 10.1038/ncomms11419

**Published:** 2016-05-09

**Authors:** Marco Tomamichel, Mario Berta, Joseph M. Renes

**Affiliations:** 1 School of Physics, University of Sydney, Sydney, New South Wales 2006, Australia; 2 Institute for Quantum Information and Matter, Division of Physics, Mathematics and Astronomy, California Institute of Technology, Pasadena, California 91125, USA; 3 Department of Physics, Institute for Theoretical Physics, ETH Zurich, 8093 Zürich, Switzerland

## Abstract

The quantum capacity of a memoryless channel determines the maximal rate at which we can communicate reliably over asymptotically many uses of the channel. Here we illustrate that this asymptotic characterization is insufficient in practical scenarios where decoherence severely limits our ability to manipulate large quantum systems in the encoder and decoder. In practical settings, we should instead focus on the optimal trade-off between three parameters: the rate of the code, the size of the quantum devices at the encoder and decoder, and the fidelity of the transmission. We find approximate and exact characterizations of this trade-off for various channels of interest, including dephasing, depolarizing and erasure channels. In each case, the trade-off is parameterized by the capacity and a second channel parameter, the quantum channel dispersion. In the process, we develop several bounds that are valid for general quantum channels and can be computed for small instances.

## Abstract (web summary)

The maximal rate at which quantum information can be reliably communicated through many uses of a memoryless quantum channel is determined by its quantum channel capacity. Here, the authors demonstrate that such an asymptotic characterization is insufficient in practical scenarios.

## Graphical abstract

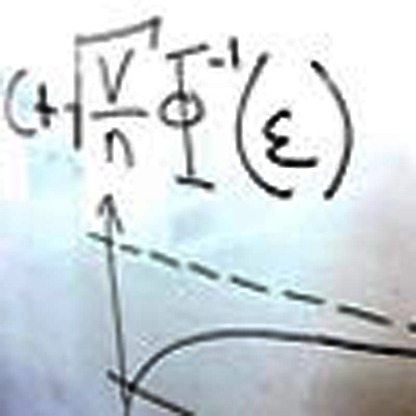

One of the quintessential topics in quantum information theory is the study of reliable quantum information transmission over noisy quantum channels. Here ‘channel' simply refers to a description of a physical evolution. In the standard formulation, one considers communication between two points connected by a memoryless channel that can be used many times in sequence. In this case, the sender first encodes a quantum state into a sequence of registers and then sends them one by one through the channel to the receiver. The receiver collects these registers and then attempts to decode the quantum state. Equivalently, one considers a collection of physical qubits that are exposed to independent noise. The goal is then to encode quantum information (logical qubits) into this system (physical qubits) so that the quantum information can be retrieved with high fidelity after a given time. One of the primary goals of information theory is to find fundamental limits imposed on any coding scheme that attempts to accomplish this task.

Following a tradition going back to Shannon's groundbreaking work^1^, this problem is usually studied asymptotically: the quantum capacity of a channel^2^,^3^,^4^,^5^,^6^,^7^ is defined as the optimal rate (in qubits per use of the channel) at which one can transmit quantum information with vanishing error as the number of sequential channel uses increases to infinity. In the context of information storage, the rate simply corresponds to the ratio of logical to physical qubits, and the number of physical qubits is taken to be asymptotically large. Such an asymptotic analysis has proven to be pertinent in the analysis of classical communication (cc) systems—but is it also satisfactory in the quantum setting?

Achieving (or approximately achieving) the quantum capacity generally requires both the receiver and sender to coherently manipulate an array of qubits that grows proportionally with the number of channel uses. More precisely, the sender is required to prepare arbitrary states that are entangled between all channel inputs and the receiver needs to perform a joint measurement on all channel outputs. While classical computers can readily operate on very large amounts of data, at least for the near future it appears unrealistic to expect that encoding and decoding circuits can store or coherently manipulate large numbers of qubits. Thus, it is natural to ask how well quantum coding schemes perform when we restrict the size of the quantum devices used for encoding the channel inputs and decoding its outputs. This is equivalent to considering communication with only a fixed number of channel uses.

In this work, following the footsteps of recent progress in classical information theory^8^,^9^,^10^,^11^, we investigate how well one can transmit quantum information in a realistic scenario where the number of channel uses is limited. The quantum capacity is at most a proxy for the answer to this question, and we show with concrete examples that it is often not a very good one. For example, we find that in the order of a 1,000 qubits are required to get within 90% of the quantum capacity of a typical qubit dephasing channel. To overcome this issue, we develop a more precise approximate characterization of the performance of optimal coding schemes that takes into account finite size effects. We find that these effects are succinctly described by a second channel parameter (besides its capacity), which we name quantum channel dispersion. As such, our work generalizes recent progress in the study of cc over quantum channels^12^,^13^.

## Results

### Model for quantum communication

**Figure 1:**
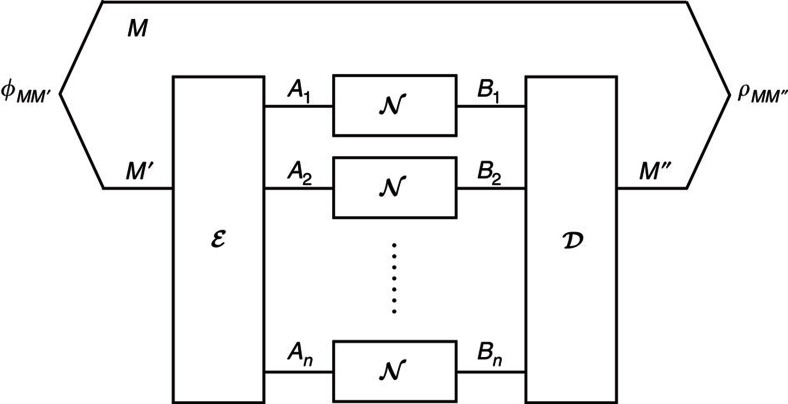
Coding scheme for entanglement transmission. Coding scheme for entanglement transmission over *n* uses of a channel 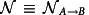. The systems *M*, *M*′ and *M*″ are isomorphic. The encoder 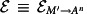 encodes the part *M*′ of the maximally entangled state *φ*_*MM*′_ into the channel input systems. Later, the decoder 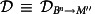 recovers the state from the channel output systems. The performance of the code is measured using the fidelity *F*(*φ*_*MM*″_, *ρ*_*MM*″_).

In this work, we focus on codes enabling a state entangled with a reference system to be reliably transmitted through the channel. This is a strong requirement: reliable entanglement transmission implies reliable transmission, on average, of all pure input states. The coding scheme is depicted in Fig. 1. We are given a quantum channel 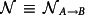 and denote by 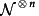 the *n*-fold parallel repetition of this channel. An entanglement-transmission code for 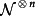 is given by a triplet 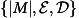, where |*M*| is the local dimension of a maximally entangled state 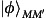 that is to be transmitted over 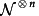. The quantum channels 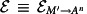 and 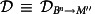 are encoding and decoding operations, respectively. With this in hand, we now say that a triplet {*R*, *n*, *ɛ*} is achievable on the channel 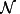 if there exists an entanglement-transmission code satisfying


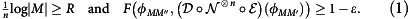


Here *R* is the rate of the code, *n* is the number of channel uses and *ɛ* is the tolerated error or infidelity, measured in terms of Uhlmann's fidelity^14^, 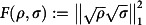.

The non-asymptotic achievable region of a quantum channel 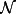 is then given by the union of all achievable triplets {*R*, *n*, *ɛ*}. The goal of (non-asymptotic) information theory is to find tight bounds on this achievable region, in particular to determine if certain triplets are outside the achievable region and thus forbidden. For this purpose, we define its boundary


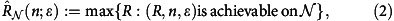


and investigate it as a function of *n* for a fixed value of *ɛ*. We will often drop the subscript 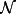 if it is clear which channel is considered. An alternative approach would be to investigate the boundary 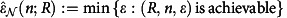, as in ref. 15. This leads to the study of error exponents (and the reliability function), as well as strong converse exponents. We will not discuss this here since such an analysis usually does not yield good approximations for small values of *n*.

To begin, let us rephrase the seminal capacity results in this language. The quantum capacity is defined as the asymptotic limit of 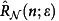 when *n* (first) goes to infinity and *ɛ* vanishes. The capacity can be expressed in terms of a regularized coherent information^2^,^3^,^5^,^6^,^7^,^16^:


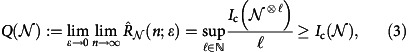


where the coherent information *I*_c_ is an entropic functional defined in Methods. This result is highly unsatisfactory, not least because the regularization makes its computation intractable. (The supremum in equation (3) is necessary in the following sense: there does not exist a universal constant 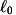 such that 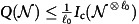 for all channels 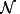
17.) Worse, the statement is not as strong as we would like it to be because it does not give any indication of the fundamental limits for finite *ɛ* or finite *n*.

For example, even sticking to the asymptotic limit for now, we might be willing to admit a small but nonzero error in our recovery. Formally, instead of requiring that the error vanishes asymptotically, we only require that it does not exceed a certain threshold, *ɛ*. Can we then achieve a higher asymptotic rate in the above sense? For cc this is ruled out by Wolfowitz's strong converse theorem^18^. However, surprisingly, the answer to this question is not known for general quantum channels. Recent work^19^ at least settles the question in the negative for a class of generalized dephasing channels and in particular for the qubit dephasing channel


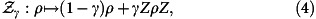


where *γ*∈[0, 1] is a parameter and *Z* is the Pauli *Z* operator. Dephasing channels are particularly interesting examples because dephasing noise is dominant in many physical implementations of qubits. The results of ref. 19 thus allow us to fully characterize the achievable region in the limit *n*→∞ for such channels, and in particular ensure that


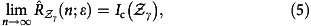


independent of the value of *ɛ*∈(0, 1). Note also that the regularization is not required here since dephasing channels are degradable^20^.

Here we go beyond studying the problem in the asymptotic limit and develop characterizations of the achievable region for finite values of *n*. We find inner (achievability) and outer (converse) bounds on the boundary of the achievable region. We first discuss these bounds for three important example channels, the qubit dephasing, erasure and depolarizing channel, and then present bounds for general channels.

### Qubit dephasing channel

We show that the non-asymptotic achievable region of the qubit dephasing channel is equivalent to the corresponding region of a (classical) binary symmetric channel. This allows us to employ results from classical information theory^10^,^21^,^22^ to establish the following characterization of the achievable region for the qubit dephasing channel.

Theorem 1. For the qubit dephasing channel 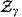 with *γ*∈[0, 1], the boundary 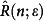 satisfies


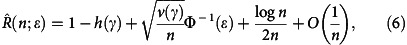


where Φ is the cumulative normal distribution function, Φ^−1^ its inverse, *h*(·) denotes the binary entropy, and *v*(·) the corresponding variance, 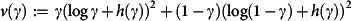.

**Figure 2:**
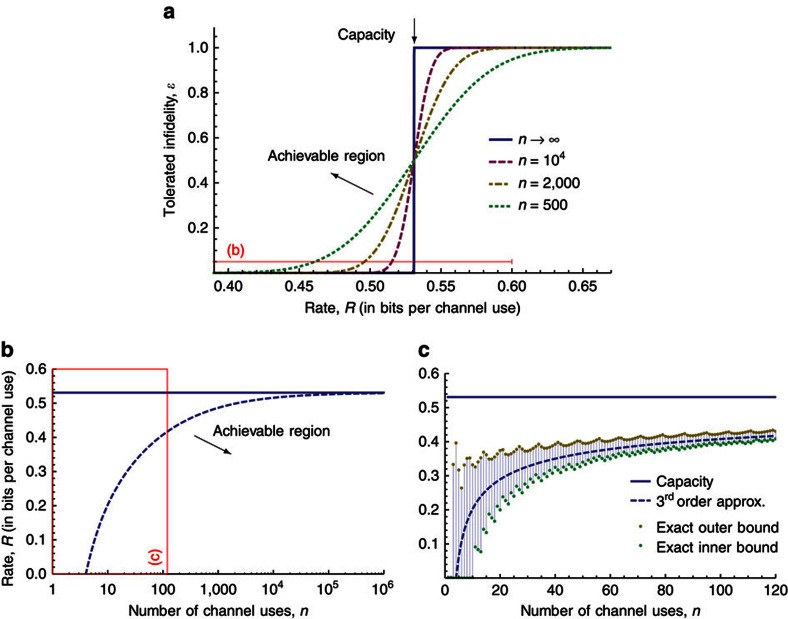
Example 1—qubit dephasing channel. Approximation of the non-asymptotic achievable rate region of a qubit dephasing channel with *γ*=0.1 (see Theorem 1). All numerical results are evaluated using the binary logarithm, that is, log≡log_2_. (**a**) Boundary of the achievable region for fixed *n* with different values (second order approximation). (**b**) Boundary of the achievable region for fixed infidelity *ɛ*=5% (third order approximation) in equation (6). (**c**) Comparison of strict bounds with third order approximation for fixed *ɛ*=5%.

The expression without the remainder term 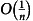 is called the third order approximation of the (boundary of the) non-asymptotic achievable region. The quantity *v*(*γ*) is the quantum channel dispersion and characterizes the finite size effects for quantum communication over the qubit dephasing channel. The approximation is visualized in Fig. 2 for an example channel with *γ*=0.1. In Fig. 2a, we plot the smallest achievable error *ɛ* as a function of the rate *R*. Here we use the second order expansion without the term 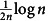 since it can conveniently be solved for *ɛ*. In the limit *n*→∞, we see an instantaneous transition of *ɛ* from 0 to 1, the signature of a strong converse: coding below the capacity 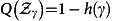 is possible with perfect fidelity, whereas coding above the capacity will necessarily result in a vanishing fidelity.

In Fig. 2b, we plot the third order approximation in equation (6) for the highest achievable rate, 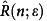, as a function of *n* for a fixed fidelity of 95% (that is, we set *ɛ*=5%). For example, this allows us to calculate how many times we need to use the channel to approximately achieve the quantum capacity. The third order approximation shows that we need ∼850 channel uses to achieve 90% of the quantum capacity. Note that a coding scheme achieving this would probably require us to coherently manipulate 850 qubits in the decoder, which appears to be a quite challenging task. This example shows that the capacity does not suffice to characterize the ability of a quantum channel to transmit information, and further motivates the study of the achievable region for finite *n*.

Finally, we remark that the third order approximation is quite strong even for small *n*. To prove this, we compare it to exact upper and lower bounds on 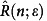 in Fig. 2c and see that the remainder term 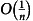 becomes negligible for fairly small *n*≈100 for the present values of *γ* and *ɛ*.

### Qubit erasure channel

Another channel we can analyse in this manner is the qubit erasure channel, given by the map


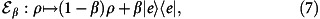


where *β*∈[0, 1] is the probability of erasure and |*e*〉〈*e*| is a pure state orthogonal to *ρ* that indicates erasure. Here we investigate coding schemes that allow free cc assistance between the sender and receiver in both directions, in parallel to the quantum transmission. This setting is quite natural because we can often assume that cc is considerably easier to implement than quantum communication (see Fig. 5 in Methods for a description of such codes). We denote the corresponding boundary of the achievable region by 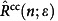. Since this includes all codes that do not take advantage of cc, we clearly have 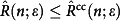 for all channels. This inequality is strict for the erasure channel but for the dephasing channel we find that the asymptotic expansion in equation (6) holds for both 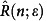 and 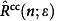, that is, cc assistance does not help asymptotically (up to third order).

For the qubit erasure channel, we can determine the boundary 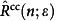 exactly, again by generalizing^19^ and relating the problem to that of the classical erasure channel.

Theorem 2. For the qubit erasure channel 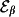 with *β*∈[0, 1], the boundary 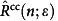 satisfies


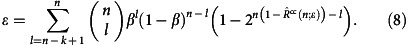


Moreover, for large *n*, we have the expansion


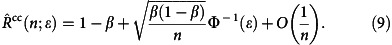


**Figure 3:**
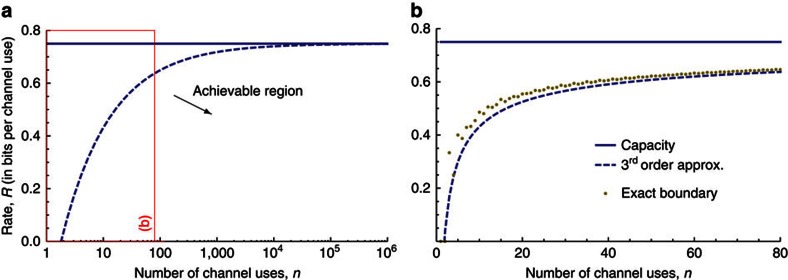
Example 2—qubit erasure channel. Approximation of the non-asymptotic achievable rate region with classical communication assistance of a qubit erasure channel with *β*=0.25 and fixed infidelity *ɛ*=1% (see Theorem 2). (**a**) Boundary of the achievable region. (**b**) Comparison of exact bounds with third order approximation for small values of *n*.

The latter expression is a third order approximation of the achievable region, where 1−*β* is the quantum capacity and *β*(1−*β*) is the quantum channel dispersion of the qubit erasure channel. In Fig. 3, we show this approximation for a qubit erasure channel with *β*=0.25 and fidelity 99%. In Fig. 3a, we see that the non-asymptotic achievable region reaches 90% of the channel capacity for *n*≈180. Again, this confirms that the non-asymptotic treatment is crucial in the quantum setting. In Fig. 3b, we compare the third order approximation with the exact boundary of the achievable region in equation (8). We see that the approximation is already very precise (and the term 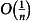 thus negligible) for fairly small *n*≈50.

### Qubit depolarizing channel

Another prominent channel is the qubit depolarizing channel. It is given by the map


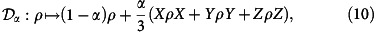


where *α*∈[0, 1] is a parameter and *X*, *Y*, *Z* are the Pauli operators. For this channel, no closed formula for the quantum capacity 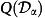 is known, and the coherent information


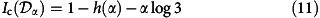


is only a strict lower bound on it^23^. However, various upper bounds on the quantum capacity of the qubit depolarizing channel have been established^24^,^25^,^26^,^27^,^28^. For example, in (ref. 24, Theorem 2) it is essentially shown that 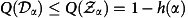, the quantum capacity of the qubit dephasing channel with dephasing parameter *α*. Here we extend this result to the non-asymptotic setting and find the following outer (converse) bound for the achievable rate region that holds even with cc assistance.

Theorem 3. For the qubit depolarizing channel 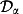 with *α*∈[0, 1], the boundary 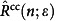 satisfies


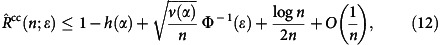


where the right-hand side is simply the asymptotic expansion of the boundary of the achievable rate region for the qubit dephasing channel 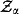 with dephasing parameter *α* as in Theorem 1.

**Figure 4:**
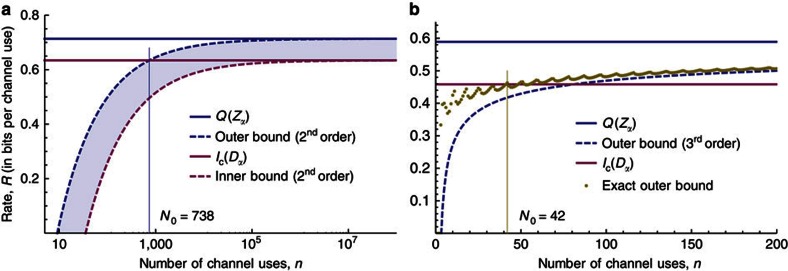
Example 3—qubit depolarizing channel. Approximate inner and outer bounds on the non-asymptotic achievable rate region for the depolarizing channel (see Theorems 3 and 5) for fixed tolerated infidelity *ɛ*. The outer bounds apply to codes with classical communication assistance, whereas the inner bounds consider only unassisted codes. (**a**) Inner and outer bounds for *α*=0.05 and *ɛ*=1%. (**b**) Exact outer bound for *α*=0.0825 and *ɛ*=5.5%.

In Fig. 4a, we plot the second order approximation of the outer bound for a depolarizing channel with *α*=0.05 and 99% fidelity. We see that to implement a code with a communication rate that exceeds the coherent information equation (3), we will need a quantum device that can process at least *N*_0_=738 qubits coherently. Moreover, this statement remains true even if we allow for codes with cc assistance. This indicates that the question of whether the coherent information is a good or bad lower bound on the asymptotic quantum capacity is not of immediate practical relevance as long as we do not have a quantum computer that is able to perform a decoding operation on many hundreds of qubits.

In Fig. 4b, we examine a qubit depolarizing channel with parameters *α*=0.0825 and *ɛ*=5.5%. Instead of using an approximation for the outer bound, we use the exact outer bound to give the answer (it is 42) to the question of how many channel uses we need at minimum to exceed the coherent information. However, note that this does not give us any indication of what code (in particular if it is assisted or not), if any, can achieve this point.

### General outer and inner bounds

We have so far focused our attention on three specific (albeit very important) examples of channels. However, many of the results derived in this article also hold more generally. For example, we find the following outer (converse) bound.

Theorem 4. For any quantum channel 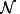, the boundary 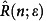 satisfies


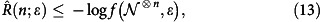


where 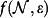 is the solution to a semidefinite optimization programme defined in equation 24 and Methods. Moreover, if 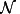 is covariant, we find the asymptotic expansion


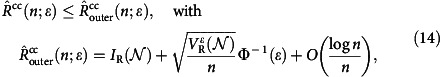


where the Rains information, 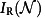, and its variance, 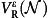, are entropic functionals defined in equation (28) and equation (29) and Methods.

In fact, the bound in equation (13) holds also for codes that allow classical post-processing (cpp), as discussed in the Supplementary Notes. Covariant channels are discussed in Methods, and include the dephasing, erasure and depolarizing channels treated above. The semidefinite optimization programme 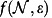 is similar in spirit to the metaconverse for classical coding^10^,^29^,^30^. For quantum coding, alternative semidefinite optimization programme lower bounds on the error boundary 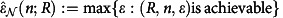 for fixed rate *R* have been derived in ref. 15. Note that our bound equation (14) is tight up to the second order asymptotically for the qubit dephasing channel (Theorem 1) and the erasure channel with cc assistance (Theorem 2). However, in the generic covariant case the bound is not expected to be tight. Moreover, if the channel is not covariant we cannot asymptotically expand our outer bounds on the achievable rate region in a closed form as above.

Finally, an inner (achievability) bound of the form shown in Theorem 1 also holds generally for all quantum channels.

Theorem 5. For any quantum channel 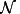, the boundary 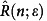 satisfies


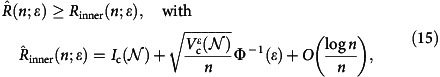


where the coherent information, 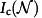, and its variance, 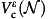, are entropic functionals defined in equation 35 and equation 36 and Methods.

Note that the bound equation (15) is tight up to the second order asymptotically for the qubit dephasing channel (Theorem 1). For the erasure channel, this bound does not match the outer bound since it does not take into account cc assistance. For general channels, the bound does not tightly characterize the achievable region. In particular, for *n*→∞, it converges to the coherent information and not the regularized coherent information, which can be strictly larger^23^. However, we have reasons to conjecture that the bound is tight for degradable channels^20^,^31^.

The same inner bound has been shown independently and concurrently in ref. 32 using a different decoder.

## Discussion

The main contributions of this work can be summarized as follows. We showed—both analytically and quantitatively—that the quantum channel capacity is insufficient to characterize achievable communication rates in the finite resource setting. We provided a remedy, showing that the capacity and quantum channel dispersion together provide a very good characterization, in particular for the practically relevant qubit dephasing, depolarization and erasure channels. This is crucial for practical considerations where one would like to rely on a simple and easy to evaluate formula to estimate the achievable rate region. For instance, one can use the estimated optimal rate region to benchmark explicit codes, for example, in designing a quantum repeater.

More precisely, for general channels, we gave inner (achievability) and outer (converse) bounds on the boundary of the achievable region for quantum communication with finite resources (*cf.,* Theorems 5 and 4). These bounds can be formulated as semidefinite programmes and thus evaluated for small instances. For larger instances, we show that the bounds admit a second order approximation featuring the dispersion (for the converse bound this requires the assumption of channel covariance) which can be evaluated efficiently. We then showed that the inner and outer bounds agree for the qubit dephasing channel (*cf.,* Fig. 2) and qubit erasure channel with cc assistance (*cf.,* Fig. 3) up to the third order asymptotically. For the qubit depolarizing channel (*cf.,* Fig. 4), we gave separate second order approximations for the inner and outer bounds. Closing the gap between these bounds (see shaded area in Fig. 4a), even asymptotically, remains one of the most tantalizing open questions in quantum information theory^26^.

For general channels, many questions remain open. For example, we would like to understand if the inner bound in Theorem 5 characterizes the achievable region for all degradable channels^20^ (*cf.,* the open questions in ref. 19). Also it would be interesting to explore higher order refinements for channels with zero quantum capacity (for example, for the erasure channel with *β*≥1/2 and no assistance). This might lead to a better understanding of superactivation of the quantum capacity^33^. Taking a broader view, convex relaxation, such as our semidefinite programme, provides a promising approach to better understand the rate region beyond studying entropic properties. For practical applications, the most important channel not addressed here is the qubit amplitude damping channel, and it is an important open question to analyse it in the finite resource regime.

Finally, we note that our analysis can be extended to the case of entanglement-assisted quantum communication. A short exhibition of this extension is provided in Supplementary Note 1.

## Methods

### General notation and codes

Here we sketch the main ideas of the proofs of Theorems 4 and 5, and a more detailed exposition is given in Supplementary Note 2. A detailed analysis of the example channels in Theorems 1–3 can be found in Supplementary Note 3.

We denote finite-dimensional Hilbert spaces corresponding to individual quantum systems by capital letters. In particular, we use *A* and *B* to model the channel input and output space, respevtively, whereas *M* and the isomorphic spaces *M*′ and *M*″ are used to model the quantum systems containing the maximally entangled state to be transmitted. We also use *A*^*n*^ to denote the *n*-fold tensor product of *A* for any 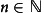. We use 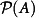 to denote the set of positive semidefinite operators on *A*, and 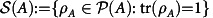 to denote quantum states with unit trace on *A*. We denote the dimension of *A* by |*A*|. Pure states are of the form 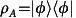, where 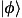 is a vector in *A* and 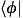 its dual functional. The marginals of a bipartite quantum state 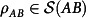 on *A* and *B* are denoted by *ρ*_*A*_ and *ρ*_*B*_, respectively. A quantum channel 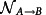 is a completely positive trace-preserving map from states on *A* to states on *B*. For any state *ρ*_*A*_, we define the canonical purification 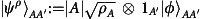, where *A*′ is isomorphic to *A* and *φ*_*AA*′_ is the maximally entangled state. To express our results, we use Umegaki's quantum relative entropy^34^, 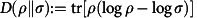 and the quantum relative entropy variance^35^,^36^, 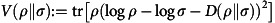. The coherent information and the coherent information variance^35^ of a bipartite state *ρ*_*AB*_ are given as


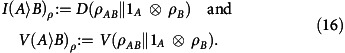


**Figure 5:**
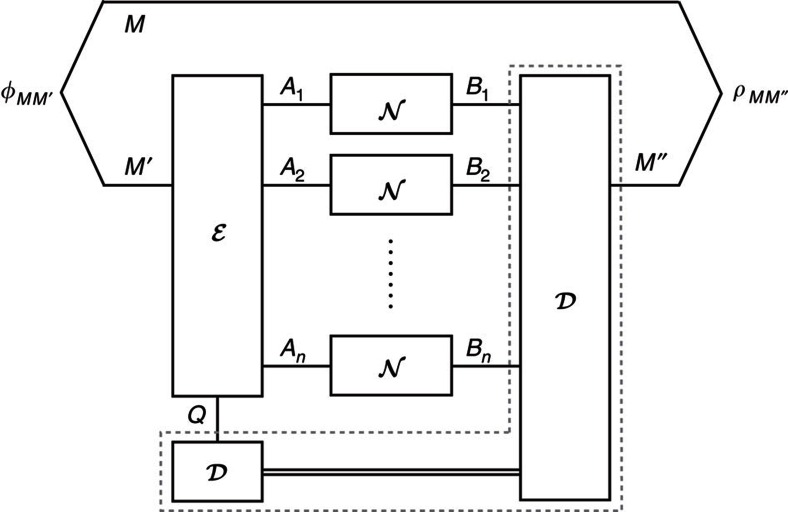
Coding scheme for entanglement transmission with classical post-processing. Coding scheme for entanglement transmission over *n* uses of a channel 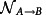 with classical post-processing. The encoder 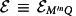 encodes *M*' into the channel input systems and a local memory *Q*. Later, the decoder 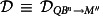 recovers the maximally entangled state from the channel output systems and the memory *Q* using classical communication and local operations. The performance of the code is measured using the fidelity 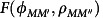.

We have defined unassisted entanglement-transmission codes in Results. Let us reintroduce them in the context of codes assisted by cpp. For this, we consider any quantum channel 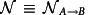 and its *n*-fold extension 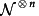 that maps states on *A*^*n*^ to states on *B*^*n*^. An entanglement-transmission code assisted by cpp for 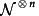 is given by a triplet 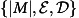, as depicted in Fig. 5. Here |*M*| is the local dimension of a maximally entangled state 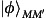 that is to be transmitted over 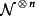. The encoder 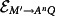 is a completely positive trace-preserving map that prepares the channel inputs *A*_1_, *A*_2_, … *A*_*n*_ and a local memory system, which we denote by *Q*. The decoder 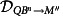 is a completely positive trace-preserving map that is restricted to local operations and cc with regard to the bipartition *Q*:*B*^*n*^ and outputs *M*″ on the receiver's side.

The boundary of the achievable rate region for these codes is denoted by 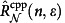. Finally, we note that unassisted codes are recovered if we choose *Q* to be trivial. Hence, unassisted codes are contained in the set of assisted codes and we have 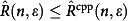. Moreover, for covariant channels we will see later that 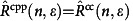 since all cc can be postponed to after the quantum communication. Hence, while we will in the following derive our converse bounds for 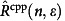, they are also valid for 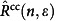 when the channel is covariant.

### Outer bounds on the achievable rate region

Our converse results are inspired by the strong converse results for generalized dephasing channels and the metaconverse for classical channel coding^10^. They are expressed in terms of the channel hypothesis testing Rains relative entropy, which is defined following the generalized divergence framework discussed in ref. 19. First, let us introduce the Rains set^25^,^37^, which is a superset of the set of positive partial transpose (PPT) states. It is defined as 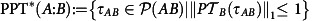, where 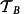 denotes the partial transpose map on *B*. We have the following crucial inequality (ref. 38, Lemma 2): for every *σ*_*AB*_∈PPT*(*A*:*B*), we have


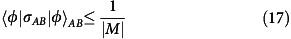


for all maximally entangled states *φ*_*AB*_ of local dimension |*M*|. The set is closed under local quantum operations on *A* and *B* supported by cc between *A* and *B*. Finally, we employ the hypothesis testing relative entropy^39^, (in the form of ref. 40)


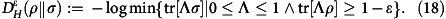


We first formulate a general metaconverse bounding possible rates *R* given a tolerated infidelity *ɛ* for a single use (*n*=1) of a fixed channel 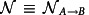. For this purpose, consider any state 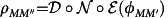 at the output of a code achieving fidelity 1−*ɛ* and any state *σ*_*MM*″_∈PPT*(*M*:*M*″). These must satisfy, according to equation (17),


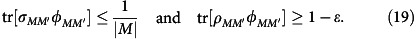


From this, we can conclude that 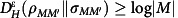 by using the projection Λ=*φ*_*MM*′_ as our hypothesis test in equation (18). At this stage, we can use the data-processing inequality of the hypothesis testing divergence^40^ to remove the decoder from the picture. Minimizing over all auxiliary states *σ*_*MQB*_∈PPT*(*MQ*:*B*), this yields


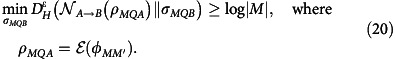


Crucially, we rely on the fact that PPT*(*MQ*:*B*) gets mapped into PPT*(*M*:*M*″) by the action of the decoder. Now we observe that by choosing the register *Q* sufficiently large, we can assume that the encoder is an isometry without loss of generality. Hence, for a fixed marginal *ρ*_*A*_=tr_*QM*_(*ρ*_*MQA*_), we can rewrite the above inequality using the substitutions *A*→*A*′ and *MQ*→*A* as


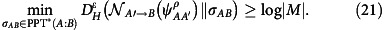


Optimized over all codes (and thus marginals *ρ*_*A*_), we find that


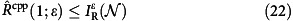


with the channel hypothesis testing Rains relative entropy defined as


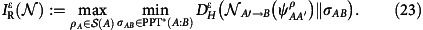


Note that this outer bound also holds for coding schemes with (unphysical) PPT assistance including classical pre- and post-processing assistance (see ref. 15 for a more comprehensive discussion of PPT assisted codes). The bound can be further relaxed to 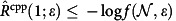, where 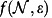 is a semidefinite programme given below. This semidefinite optimization is discussed in more detail in Supplementary Note 4.


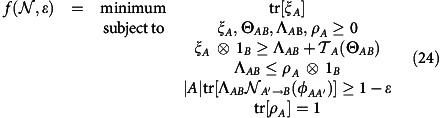


Moreover, the bound in equation (22) has the useful property that channel symmetries can be used to simplify its form, as we will see next. Suppose *G* is a group represented by unitary operators *U*_*g*_ on *A* and *V*_*g*_ on *B*. A quantum channel 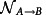 is covariant with respect to this group (and its representations) when


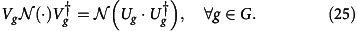


Now the main workhorse to simplify our outer bounds for channels with symmetries is (ref. 19, Proposition 2), which states that we may restrict the optimization in equation (23) to input states that are invariant under the rotations 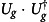 for any *g*∈*G*. For channels of the form 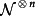 which are invariant under permutation of the input and output systems, this allows us to restrict attention to input states that are permutation invariant.

Moreover, we call a channel covariant if it is covariant with respect to a group which has a representation *U*_*g*_ on *A* that is a one-design, that is, the map 
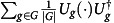
 always outputs the fully mixed state. In this case, the channel input state can be chosen to be fully mixed (respectively its purification is maximally entangled). Moreover, any such group allows for a corresponding teleportation protocol^41^ (see the construction in ref. 42), and thus all interactive cc can be postponed until after the quantum communication is completed by the argument given in refs 43, 44. From this, we can conclude that 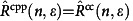 for all covariant channels.

Now let 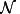 be a covariant quantum channel and *φ*_*AA*′_ a maximally entangled state. Then, our bound in equation (22) applied to the channel 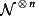 yields


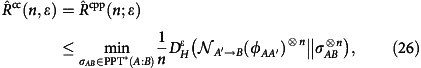


where we voluntarily restricted the minimization to product states of the form 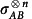 for some *σ*_*AB*_∈PPT*(*A*:*B*). Moreover, since these states have tensor power structure, the outer bound can be expanded using^35^,^36^


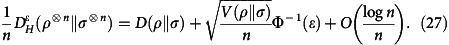


This leads to the formal statement of Theorem 4.

*Formal Theorem 4*. Let 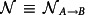 be a covariant quantum channel and let *φ*_*AA*′_ be maximally entangled. We define the Rains information of 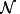 as


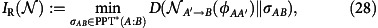


where we let Π⊂PPT*(*A*:*B*) be the set of states that achieve the minimum. The variance of the channel Rains information is


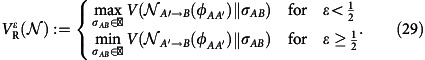


For any fixed *ɛ*∈(0, 1), the achievable region with classsical communication assistance satisfies


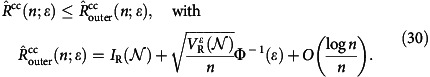


### Inner bounds on the achievable rate region

We use the decoupling approach^45^,^46^,^47^, and in particular a one-shot bound^31^ which is a tighter version of previous bounds^48^,^49^,^50^. To reproduce their result, we need the following additional notation. Sub-normalized quantum states are collected in the set 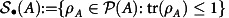. The purified distance^51^ *ɛ*-ball around 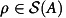 is then defined as 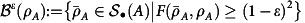. Finally, for 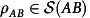 and *ɛ*≥0 the smooth conditional min-entropy^51^,^52^,^53^ is defined as


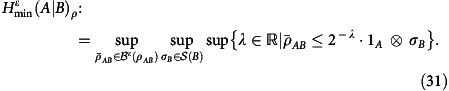


Let us now restate (Proposition 20 in ref. 31) expressed in terms of the non-asymptotic achievable region as introduced in the Results. Let 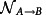 be a quantum channel with complementary channel 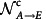. Then {*R*, 1, *ɛ*} is achievable if, for some *η*∈(0, *ɛ*] and some state 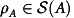, we have


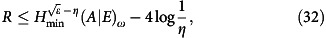


where 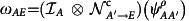. This leads immediately to the following inner bound on the achievable region. Using 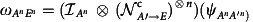, we have


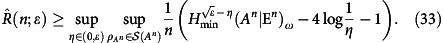


The problem with this bound is that it is generally hard to evaluate, even for moderately large values of *n*. Hence we are interested to further simplify the expression on the right-hand side in this regime. To do so, we choose 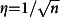 and use input states of the form 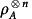. This yields the following relaxation, which holds if 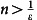:


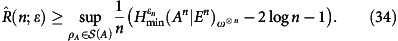


Here we introduced 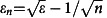 and *ω*_*AE*_ as in equation (32). Using a second order expansion^35^ similar to the one in equation (27), we give an asymptotic expansion of the expression on the right-hand side of equation (34). This yields Theorem 5.

*Formal Theorem 5*. Let 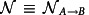 be a quantum channel. We define its coherent information as


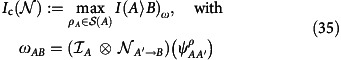


and let 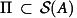 be the set of states that achieve the maximum. Define


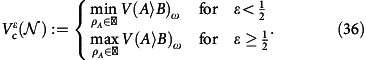


Then, for any fixed *ɛ*∈(0, 1), the achievable region satisfies


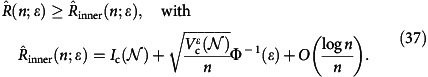


## Additional information

**How to cite this article:** Tomamichel, M. *et al*. Quantum coding with finite resources. *Nat. Commun.* 7:11419 doi: 10.1038/ncomms11419 (2016).

## Supplementary Material

Supplementary Information Supplementary Figure 1, Supplementary Notes 1-4 and Supplementary References
