# Supplementary material for: Quantum coding with finite resources
Source: Nat Commun. 2016 May 9;7:11419. doi: 10.1038/ncomms11419 (PMC4865765; doi:10.1038/ncomms11419)
Supplement: Supplementary Information — Supplementary Figure 1, Supplementary Notes 1-4 and Supplementary References [file ncomms11419-s1.pdf]

Supplementary Figures

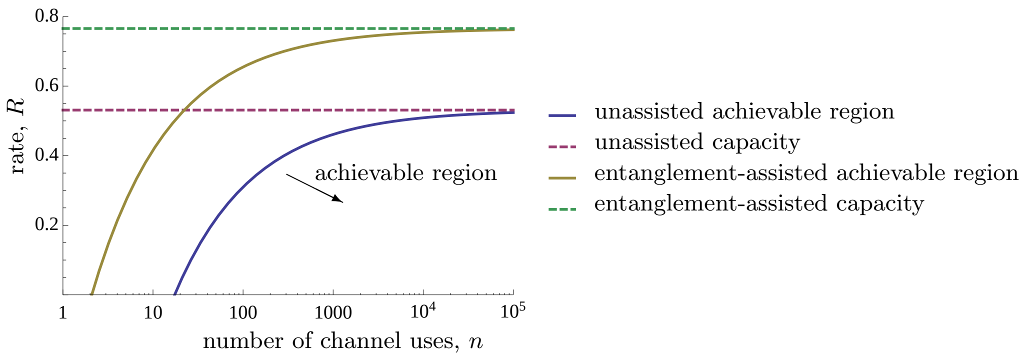

Supplementary Figure 1: **Second order approximation for a qubit dephasing channel with entanglement assistance.** Second order approximation of the achievable region (with and without entanglement assistance) for a qubit dephasing channel with $\varepsilon = 1\%$ and $\gamma = 0.1$. The achievable region is enlarged in the presence of entanglement, as discussed in Supplementary Note 1.

Supplementary Notes

Supplementary Note 1: Entanglement-Assisted Communication

Finally, we would like to note that the recent results about finite resource entanglement assisted classical communication [1] can be transformed to entanglement assisted quantum communication (and this then also gives outer bounds on the achievable rate region for unassisted codes). This is accomplished by using the equivalence results in [16, App. B] which make use of quantum teleportation and superdense coding. In particular, one finds that for covariant channels $\mathcal{N}$ (which includes the qubit dephasing channel and the erasure channel) the boundary of the entanglement assisted achievable region $\hat{R}^{\mathrm{E}}(n;\varepsilon)$ satisfies

$$\hat{R}^{\mathrm{E}}(n;\varepsilon) = \frac{I(\mathcal{N})}{2} + \sqrt{\frac{V_\varepsilon(\mathcal{N})}{4n}}\Phi^{-1}(\varepsilon) + O\left(\frac{\log n}{n}\right) , \tag{1}$$

with the mutual information of the channel, $I(\mathcal{N})$, and its variance, $V^\varepsilon(\mathcal{N})$, as defined in [1]. As an example, we mention again the qubit dephasing channel $\mathcal{Z}_\gamma$ for which

$$\hat{R}^{\mathrm{E}}(n;\varepsilon) = 1 - 2h(\gamma) + \sqrt{\frac{v(\gamma)}{4n}}\Phi^{-1}(\varepsilon) + O\left(\frac{\log n}{n}\right) . \tag{2}$$

where $h(\gamma)$ denotes the binary entropy and $v(\gamma)$ the corresponding variance as defined in Theorem 1 in the main text.

The entanglement-assisted achievable region is compared with the unassisted achievable region in Supplementary Figure 1.

Supplementary Note 2: Analysis of General Channels

In this note we detail the derivations of Theorems 4 and 5 in the main document.

Outer Bounds on the Achievable Rate Region

Let us first give a self-contained proof of our outer bound for one use of the channel, using the notation introduced in the main document. Let us restate [2, Lemma 2]: For every $\sigma_{AB} \in \mathrm{PPT}^*(A:B)$, we have

$$\langle \phi | \sigma_{AB} | \phi \rangle_{AB} \leq \frac{1}{|M|} \tag{3}$$

for all maximally entangled states ϕ_{AB} of local dimension $|M|$.

Lemma 1. *Let $\mathcal{N}_{A\to B}$ be a quantum channel. Then, for any fixed $\varepsilon \in (0,1)$, the achievable region with cpp-assistance satisfies*

$$\hat{R}^{\mathrm{cpp}}(1;\varepsilon) \leq I_R^{\varepsilon}(\mathcal{N}) \,. \tag{4}$$

Proof of Lemma 1. First, observe that the encoding operation $\mathcal{E}_{M'\to AQ}$ can be chosen to be an isometry without loss of generality, because we may include any extension systems needed for the Stinespring dilation into Q. Then we may express the entanglement fidelity as follows

$$\begin{aligned} F &= \mathrm{tr}\left[\phi_{MM'} \mathcal{D}_{BQ\to M'} \circ \mathcal{N}_{A\to B} \circ \mathcal{E}_{M'\to AQ}(\phi_{MM'})\right] && (5)\\ &= \mathrm{tr}\left[\mathcal{E}_{M\to \bar{A}\bar{Q}} \otimes \mathcal{D}^{\dagger}_{BQ\to M'}(\phi_{MM'}) \mathcal{N}_{A\to B}\left(\mathcal{E}_{M'\to AQ} \otimes \mathcal{E}_{M\to \bar{A}\bar{Q}}(\phi_{MM'})\right)\right] . && (6)\end{aligned}$$

Here $\dagger$ denotes the adjoint map with regards to the Hilbert-Schmidt inner product.

Since $\mathcal{E}$ is an isometry, the state $\rho_{A\bar{A}Q\bar{Q}} = \mathcal{E}_{M'\to AQ} \otimes \mathcal{E}_{M\to \bar{A}\bar{Q}}(\phi_{MM'})$ is pure, and therefore there exists an isometry $W_{A'\to \bar{A}Q\bar{Q}}$ such that $|\rho\rangle_{A\bar{A}Q\bar{Q}} = W_{A'\to \bar{A}Q\bar{Q}} |\psi^{\rho}\rangle_{AA'}$. Thus,

$$F = \mathrm{tr}\left[W^{\dagger}_{A'\to \bar{A}Q\bar{Q}}\left(\mathcal{E}_{M\to \bar{A}\bar{Q}} \otimes \mathcal{D}^{\dagger}_{BQ\to M'}(\phi_{MM'})\right) W_{A'\to \bar{A}Q\bar{Q}} \mathcal{N}_{A\to B}(\psi^{\rho}_{AA'})\right] . \tag{7}$$

Now consider the entanglement fidelity of any $\sigma_{A'B} \in \mathrm{PPT}^*(A'\!:\!B)$ instead of $\mathcal{N}_{A\to B}(\psi^{\rho}_{AA'})$. By (3) we have

$$\mathrm{tr}\left[\phi_{MM'}\left(\mathcal{E}^{\dagger}_{M\to \bar{A}\bar{Q}} \otimes \mathcal{D}_{BQ\to M'}\left(W_{A'\to \bar{A}Q\bar{Q}} \sigma_{A'B} W^{\dagger}_{A'\to \bar{A}Q\bar{Q}}\right)\right)\right] \leq \frac{1}{M} , \tag{8}$$

as the operations on $\sigma_{A'B}$ are all PPT-preserving. We may write this bound in terms of the hypothesis-testing relative entropy, because

$$\Lambda_{A'B} := W^{\dagger}_{A'\to \bar{A}Q\bar{Q}}\left(\mathcal{E}_{M\to \bar{A}\bar{Q}} \otimes \mathcal{D}^{\dagger}_{BQ\to M'}(\phi_{MM'})\right) W_{A'\to \bar{A}Q\bar{Q}} \tag{9}$$

is a feasible test to discriminate between $\mathcal{N}_{A\to B}(\psi^{\rho}_{A'A})$ and $\sigma_{A'B}$. That is, $\Lambda_{A'B}$ satisfies $0 \leq \Lambda_{A'B} \leq 1_{A'B}$ and $\mathrm{tr}[\Lambda_{A'B}\mathcal{N}_{A\to B}(\psi^{\rho}_{A'A}))] \geq 1-\varepsilon$, the former since $\mathcal{D}$ is completely-positive and trace-preserving and $\mathcal{E}$ and W are isometries, the latter by assumption that $F \geq 1-\varepsilon$. From (8) we then obtain

$$\hat{R}^{\mathrm{cpp}}(1;\varepsilon) \leq D_H^{\varepsilon}(\mathcal{N}_{A\to B}(\psi^{\rho}_{A'A}) \,\|\, \sigma_{A'B}) \,. \tag{10}$$

Since the bound holds for all $\sigma_{A'B} \in \mathrm{PPT}^*(A'\!:\!B)$, we may take the minimum over this set. The resulting bound depends on the precise channel input $\rho_A \in \mathcal{S}(A)$ used by the code, but we can remove the dependence by taking the maximum over all possible inputs. The result is (4). □

Relaxation. We may relax the bound from Lemma 1 by restricting the form of the possible states σ_{AB} in the definition of the hypothesis testing Rains relative entropy $I_R^\varepsilon(\mathcal{N})$. For this purpose, a quantum channel $\mathcal{N}_{A\to B}$ is called PPT preserving if a PPT state input necessarily results in a PPT state output. It turns out that PPT-preserving channels output PPT states for any input, since they have PPT Choi states [3] (see the discussion after Eq. 4.13). Channels with PPT Choi states were also called PPT-binding in [4]. For our purposes here, we may consider trace non-increasing PPT-preserving channels, and write $\mathcal{M}_{A\to B} \in \mathrm{PPT}$ when the Choi state M_{AB} of $\mathcal{M}_{A\to B}$ satisfies $\mathcal{T}_A(M_{AB}) \geq 0$ and $\mathrm{tr}_B\, M_{AB} \leq 1_A$.

Corollary 2. *Let $\mathcal{N}_{A\to B}$ be a quantum channel. We define the function*

$$f(\mathcal{N},\varepsilon) := \min_{\rho_A\in\mathcal{S}(A)} \min_{\Lambda_{AB}\in\Gamma(\rho_A,\mathcal{N},\varepsilon)} \max_{\mathcal{M}_{A\to B}\in PPT} \mathrm{tr}[\Lambda_{AB}M_{AB}]\,, \tag{11}$$

with the set $\Gamma(\rho_A,\mathcal{N},\varepsilon) := \{\Lambda_{AB} : 0 \leq \Lambda_{AB} \leq \mathcal{T}_A(\rho_A)\otimes 1_B, \mathrm{tr}[\Lambda_{AB}N_{AB}] \geq 1-\varepsilon\}$, and the Choi states $M_{AB} := (\mathcal{I}_A \otimes \mathcal{M}_{A'\to B})(|A|\phi_{AA'})$ of $\mathcal{M}_{A\to B}$ and N_{AB} of $\mathcal{N}_{A\to B}$, respectively. Then, for any fixed $\varepsilon\in(0,1)$, the achievable region satisfies

$$\hat{R}^{\mathrm{cpp}}(1;\varepsilon) \leq -\log f(\mathcal{N},\varepsilon)\,. \tag{12}$$

Proof. Suppose that $\sigma_{AB} = (\mathcal{I}_A\otimes\mathcal{M}_{A'\to B})(\psi^\rho_{AA'})$ for some trace non-increasing PPT-preserving (PPT-binding) channel $\mathcal{M}_{A\to B}$. Any such σ_{AB} is in $\mathrm{PPT}^*(A:B)$, therefore we may use this choice in the right-hand side of (4), which yields

$$\hat{R}^{\mathrm{cpp}}(1;\varepsilon) \leq -\log \min_{\rho_A\in\mathcal{S}(A)} \max_{\mathcal{M}_{A\to B}\in\mathrm{PPT}} \min_{\substack{0\leq\Lambda'\leq 1\\ \mathrm{tr}[\Lambda'\mathcal{N}(\rho)]\geq 1-\varepsilon}} \mathrm{tr}\left[\Lambda'_{AB}(\mathcal{I}_A\otimes\mathcal{M}_{A'\to B})(\psi^\rho_{AA'})\right]. \tag{13}$$

Now we may define $\Lambda_{AB} = (\mathcal{T}_A(\rho_A))^{1/2}\Lambda'_{AB}(\mathcal{T}_A(\rho_A))^{1/2}$ and find

$$\hat{R}^{\mathrm{cpp}}(1;\varepsilon) \leq -\log \min_{\rho_A\in\mathcal{S}(A)} \max_{\mathcal{M}_{A\to B}\in\mathrm{PPT}} \min_{\Lambda_{AB}\in\Gamma(\rho_A,\mathcal{N},\varepsilon)} \mathrm{tr}[\Lambda_{AB}M_{AB}]\,. \tag{14}$$

Finally for fixed channel input ρ_A, we can reverse the order of the inner optimizations in (14) by von Neumann's minimax theorem, since the objective function is linear and the sets are both convex and compact. This concludes the proof of (12). □

Furthermore, $f(\mathcal{N},\varepsilon)$ can be expressed as a semidefinite optimization program that satisfies strong duality. This is discussed in Supplementary Note 4.

Symmetries. Applied to the channel $\mathcal{N}^{\otimes n}$ we immediately get for any fixed $\varepsilon\in(0,1)$,

$$\hat{R}^{\mathrm{cpp}}(n;\varepsilon) \leq \frac{1}{n} I_R^\varepsilon(\mathcal{N}^{\otimes n})\,. \tag{15}$$

This bound is generally hard to evaluate even for moderately large n. In the following we show that symmetries of the channel can further simplify the outer bounds. Suppose G is a group represented by unitary operators U_g on A and V_g on B. A quantum channel $\mathcal{N}_{A\to B}$ is covariant with respect to G when

$$V_g\mathcal{N}(\cdot)V_g^\dagger = \mathcal{N}(U_g\cdot U_g^\dagger), \quad \forall g\in G\,. \tag{16}$$

Alternatively we can also write this as an invariance of the channel

$$\mathcal{N}(\cdot) = V_g^\dagger\mathcal{N}(U_g\cdot U_g^\dagger)V_g, \quad \forall g\in G\,. \tag{17}$$

Now the main workhorse to simplify our outer bounds for channels with symmetries is [5, Prop. 2], which states that we may restrict the optimization in Lemma 1 to covariant input states. Due to the form of the hypothesis testing Rains relative entropy, we may then also choose group invariant states σ and test operators Λ to obtain the tightest bound. Note that the semidefinite optimization outer bound in Corollary 2 inherits these symmetry simplifications. For general tensor product channels, which are invariant to permutation of the inputs and outputs, this allows us to restrict attention to pure states that are permutation invariant. Moreover, if the channel is covariant, then the channel input state can be chosen to be maximally mixed.

Asymptotics. Now let $\mathcal{N}_{A\to B}$ be a covariant quantum channel and $\phi_{AA'}$ a maximally entangled state. Then, we bound

$$\hat{R}^{\mathrm{cpp}}(n;\varepsilon) \leq \min_{\sigma_{AB}\in\mathrm{PPT}^*(A:B)} \frac{1}{n} D_H^{\varepsilon}\big(\mathcal{N}_{A'\to B}(\phi_{AA'})^{\otimes n}\big\|\sigma_{AB}^{\otimes n}\big)\,, \tag{18}$$

where we voluntarily restricted the minimization to product states $\sigma_{AB}^{\otimes n}$ in $\mathrm{PPT}^*(A\!:\!B)$. Moreover, since these states have tensor product structure, the outer bound can be expanded using [6, 7]

$$\frac{1}{n} D_H^{\varepsilon}(\rho^{\otimes n}\|\sigma^{\otimes n}) = D(\rho\|\sigma) + \sqrt{\frac{V(\rho\|\sigma)}{n}}\,\Phi^{-1}(\varepsilon) + O\left(\frac{\log n}{n}\right). \tag{19}$$

This leads to the following proposition.

Proposition 3. *Let $\mathcal{N} \equiv \mathcal{N}_{A\to B}$ be a quantum channel and let $\phi_{AA'}$ be maximally entangled. We define the channel Rains information of $\mathcal{N}$ as*

$$I_R(\mathcal{N}) := \min_{\sigma_{AB}\in\mathrm{PPT}^*(A:B)} D(\mathcal{N}_{A'\to B}(\phi_{AA'})\|\sigma_{AB})\,, \tag{20}$$

where we let $\Pi \subset \mathrm{PPT}^*(A:B)$ be the set of states that achieve the minimum. The variance of the channel Rains information is

$$V_R^{\varepsilon}(\mathcal{N}) := \begin{cases} \max\limits_{\sigma_{AB}\in\Pi} V(\mathcal{N}_{A'\to B}(\phi_{AA'})\|\sigma_{AB}) & \text{for} \quad \varepsilon < \frac{1}{2} \\ \min\limits_{\sigma_{AB}\in\Pi} V(\mathcal{N}_{A'\to B}(\phi_{AA'})\|\sigma_{AB}) & \text{for} \quad \varepsilon \geq \frac{1}{2} \end{cases}. \tag{21}$$

If $\mathcal{N}$ is covariant, then for any fixed $\varepsilon \in (0,1)$, the achievable region with cpp-assistance satisfies

$$\hat{R}^{\mathrm{cpp}}(n;\varepsilon) \leq \hat{R}^{\mathrm{cpp}}_{\mathrm{outer}}(n;\varepsilon), \quad \textit{with} \quad \hat{R}^{\mathrm{cpp}}_{\mathrm{outer}}(n;\varepsilon) = I_R(\mathcal{N}) + \sqrt{\frac{V_R^{\varepsilon}(\mathcal{N})}{n}}\,\Phi^{-1}(\varepsilon) + O\left(\frac{\log n}{n}\right). \tag{22}$$

Since we are here interested in outer bounds, we are also free to chose a potentially sub-optimal $\sigma_{AB} \in \mathrm{PPT}^*(A:B)$ to further relax this bound. As we see in the discussion of the qubit dephasing channel and the erasure channel with classical post-processing assistance, the bound from Proposition 3 is tight up to the second order asymptotically.

Inner Bounds on the Achievable Rate Region

We start with the following result [8, Prop. 20]:

Lemma 4. *Let $\mathcal{N}_{A\to B}$ be a quantum channel with complementary channel $\mathcal{N}^c_{A\to E}$. Then $\{R, 1, \varepsilon\}$ is achievable if, for any $\eta \in (0, \varepsilon]$ and any state $\rho_A \in \mathcal{S}(A)$, we have*

$$R \leq H_{\min}^{\sqrt{\varepsilon}-\eta}(A|E)_\omega - 4\log\frac{1}{\eta}\,, \tag{23}$$

where $\omega_{AE} = \big(\mathcal{I}_A \otimes \mathcal{N}^c_{A'\to E}\big)(\psi^\rho_{AA'})$.

Note that the authors of [8] use the purified distance as their figure of merit whereas we use the fidelity criterion. This accounts for the square root in the smoothing parameter of the conditional min-entropy. They also state their result for the special case $n = 1$, but this can be generalized to arbitrary $n \in \mathbb{N}$ if we simply consider $\mathcal{N}^{\otimes n}_{A\to B}$ as a single channel. This leads immediately to the following inner bound on the achievable region.

Corollary 5. *Using the notation of Lemma 4 with $\omega_{A^nE^n} = \big(\mathcal{I}_{A^n} \otimes (\mathcal{N}^c_{A'\to E})^{\otimes n}\big)(\psi^\rho_{A^nA'^n})$, we have*

$$\hat{R}(n;\varepsilon) \geq \max_{\eta\in(0,\varepsilon)} \max_{\rho_{A^n}\in\mathcal{S}(A^n)} \frac{1}{n}\Big(H_{\min}^{\sqrt{\varepsilon}-\eta}(A^n|E^n)_\omega - 4\log\frac{1}{\eta} - 1\Big)\,. \tag{24}$$

The problem with this bound is that it is generally hard to evaluate, even for moderately large values of n. Hence we are interested to further simplify the expression on the right-hand side in this regime. To do so, we choose $\eta = 1/\sqrt{n}$ and use input states of the form $\rho_A^{\otimes n}$. This yields the following relaxation, which holds if $n > \frac{1}{\varepsilon}$:

$$\hat{R}(n;\varepsilon) \geq \max_{\rho_A\in\mathcal{S}(A)} \frac{1}{n}\Big(H_{\min}^{\varepsilon_n}(A^n|E^n)_{\omega^{\otimes n}} - 2\log n - 1\Big)\,. \tag{25}$$

Here we introduced $\varepsilon_n = \sqrt{\varepsilon} - \frac{1}{\sqrt{n}}$ and ω_{AE} as in Lemma 4. Using standard second order expansion methods [7], we can give an asymptotic expansion of $\hat{R}_{\text{inner}}(n;\varepsilon)$ in (25) as follows.

Proposition 6. *Let $\mathcal{N} \equiv \mathcal{N}_{A\to B}$ be a quantum channel. We define its coherent information as*

$$I_c(\mathcal{N}) := \max_{\rho_A\in\mathcal{S}(A)} I(A\rangle B)_\omega\,, \quad \textit{with} \quad \omega_{AB} = \big(\mathcal{I}_A \otimes \mathcal{N}_{A'\to B}\big)(\psi^\rho_{AA'}) \tag{26}$$

and let $\Pi \subset \mathcal{S}(A)$ be the set of states that achieve the maximum. Define

$$V_c^\varepsilon(\mathcal{N}) := \begin{cases} \min_{\rho_A\in\Pi} V(A\rangle B)_\omega & \text{for} \quad \varepsilon < \frac{1}{2} \\ \max_{\rho_A\in\Pi} V(A\rangle B)_\omega & \text{for} \quad \varepsilon \geq \frac{1}{2} \end{cases}. \tag{27}$$

Then, for any fixed $\varepsilon \in (0,1)$, the achievable region satisfies

$$\hat{R}(n;\varepsilon) \geq \hat{R}_{\text{inner}}(n;\varepsilon), \quad \textit{with} \quad \hat{R}_{\text{inner}}(n;\varepsilon) = I_c(\mathcal{N}) + \sqrt{\frac{V_c^\varepsilon(\mathcal{N})}{n}}\,\Phi^{-1}(\varepsilon) + O\left(\frac{\log n}{n}\right). \tag{28}$$

Proof. We analyze the expression in (25) using the following asymptotic expansion of the smooth conditional min-entropy [7],

$$\frac{1}{n}H_{\min}^{\varepsilon}(A^n|E^n)_{\rho^{\otimes n}} = -I(A\rangle E)_\rho + \sqrt{\frac{V(A\rangle E)_\rho}{n}}\,\Phi^{-1}(\varepsilon^2) + O\left(\frac{\log n}{n}\right). \tag{29}$$

This yields that for any $\rho_A \in \mathcal{S}(A)$, we have

$$\hat{R}(n;\varepsilon) \geq -I(A\rangle E)_\omega + \sqrt{\frac{V(A\rangle E)_\omega}{n}}\,\Phi^{-1}(\varepsilon) + O\left(\frac{\log n}{n}\right), \tag{30}$$

and then by duality of the conditional entropy we find $-I(A\rangle E)_\omega = I(A\rangle B)_\omega$. Furthermore, it is easy to verify that $V(A\rangle E)_\omega = V(A\rangle B)_\omega$ (see, e.g., [9]). We conclude the proof by choosing an optimal state $\rho_A \in \Pi$ depending on the sign of $\Phi^{-1}(\varepsilon)$. □

Supplementary Note 3: Discussion of Channel Examples

In this note we detail the derivations of Theorems 1, 2 and 3 in the main document. This specialization builds on the results that are derived in Supplementary Note 2 for general channels.

Qubit Dephasing Channel

We determine the third order asymptotic performance of the qubit dephasing channel. We do this by directly obtaining the finite block length behavior of the qubit dephasing channel from that of the classical binary symmetric channel (BSC). First, consider the converse, particularly that of (18), applied to the channel $\mathcal{Z}_\gamma^{\otimes n}$. Using the Bell states $\phi^+_{AB} = \phi_{AA'}$ and $\phi^-_{AB} = (1_A \otimes Z_{A'})\phi_{AA'}(1_A \otimes Z_{A'})$, we immediately find

$$\omega_{AB} := \mathcal{N}_{A'\to B}(\phi_{AA'}) = (1-\gamma)\phi^+_{AB} + \gamma\phi^-_{AB}\,. \tag{31}$$

Now, in (18) we are free to pick any PPT* state to obtain a bound. Pick $\sigma_{AB} = \frac{1}{2}(\phi^+_{AB} + \phi^-_{AB})$, which gives

$$\hat{R}(n;\varepsilon) \le \hat{R}^{\mathrm{cpp}}(n;\varepsilon) \le \frac{1}{n} D_H^\varepsilon\big(\omega_{AB}^{\otimes n} \big\| \sigma_{AB}^{\otimes n}\big)\,. \tag{32}$$

(The choice of σ_{AB} is equivalent to using the convex relaxation of the bound, Corollary 2, and choosing $\mathcal{M} = \mathcal{Z}_{1/2}$ in (11).) To connect to the finite block length bounds of the BSC, consider measuring both A and B in the Pauli x basis, and let X and Y be the output random variables for A and B, respectively. For the state ω_{AB}, this results in the distribution P_{XY} in which P_X is uniformly-distributed and $P[Y = X] = 1-\gamma$. For σ_{AB}, the distribution is of product form $P_X Q_Y$ with Q_Y also uniform. Moreover, the original quantum states can be reconstructed from the classical random variables X and Y by the map which outputs ϕ^+_{AB} when $X = Y$ and ϕ^-_{AB} otherwise. Therefore, the bound becomes

$$\hat{R}(n;\varepsilon) \le \frac{1}{n} D_H^\varepsilon\big(P_{XY}^{\times n} \big\| P_X^{\times n} \times Q_Y^{\times n}\big)\,, \tag{33}$$

which is precisely the bound obtained for the BSC (see [10, Thm. 26]) which is equivalent to the classical sphere-packing bound [11, Eq. 5.8.19]. This establishes the desired upper bound.

For the achievability, we may directly employ linear codes for the classical BSC to the qubit dephasing channel. Specifically, any linear $\{R, n, \varepsilon\}$ code for the BSC (which recovers the input with probability at least $1-\varepsilon$, averaged over a uniform choice of inputs), can be converted into an $\{R, n, \varepsilon\}$ Calderbank-Shor-Steane (CSS) code for entanglement transmission over the dephasing channel. This is possible since, for a linear code, the action of the channel is a mapping among the orthogonal Bell states, which is essentially a classical action.

To formalize the connection, we begin with the description of the classical linear code by its $(n - \log|M|) \times n$ parity check matrix H. Each row $r_j \in \{0,1\}^n$ defines a parity function and the codewords c_k of the code must satisfy $c_k \cdot r_j = 0$ for all j. The associated CSS code can be defined as the simultaneous $+1$ eigenspace of the "stabilizer" operators X^{r_j}, where $X^{r_j} = X^{r_{j,1}} \otimes \cdots \otimes X^{r_{j,n}}$. (Generically, a CSS code has stabilizers of both X-type, as here, and of Z-type, i.e. composed of products of Pauli Z operators.) Crucially, the action of the channel is to apply an operator of the form Z^u, with $u \in \{0,1\}^n$, according to the distribution P_U. At the output, the receiver can simultaneously determine the eigenvalues of all the of the stabilizer operators. This information is precisely equivalent to determining the value of the parity checks of the classical linear code, called

the syndrome s. Given the syndrome, the decoder of the classical code determines a guess as to the input codeword, which is equivalent to a guess $u'(s)$ of the actual channel error.

We may also utilize this algorithm (whatever its precise details) in the quantum case, and attempt to correct the error by applying $Z^{u'(s)}$. When $u'(s)$ is the true error pattern, the quantum state is properly recovered, and the entanglement fidelity is unity. On the other hand, if $u'(s)$ is incorrect, then in the worst case the action $Z^{u'(s)+u}$ is a logical operation on the code subspace, which results in a state orthogonal to the desired entangled state. Therefore, the error probability of the classical code translates directly into the entanglement fidelity of the quantum code. Thus, we may apply finite-block length bounds for linear codes, particularly the bound in [12] (see also [10, Eq. 65]). This establishes the lower bound.

Qubit Erasure Channel

For the qubit erasure channel it is not too difficult to directly derive an outer bound and an explicit coding scheme leading to an inner bound, which precisely match for all n. Let us begin with the outer bound. Again we may relate the finite block length performance to a classical coding problem, namely the classical binary erasure channel (BEC). The argument for the outer bound proceeds very similarly to the dephasing example. The optimal channel input state corresponds to the maximally entangled state $\phi_{AA'}$, and the state produced by the channel is now

$$\omega_{AB} = (1-\beta)\phi_{AB} + \beta\pi_A \otimes |e\rangle\langle e|_B \,, \tag{34}$$

where π_A denotes the maximally-mixed state. Measurement of A in the Pauli x basis and B in the basis $\{|+\rangle, |-\rangle, |e\rangle\}$ produces the distribution P_{XY} with P_X uniform and $Y = X$ with probability $1-\beta$ and $Y = e$ with probability β. The original state can be reconstructed using the map which sends (X, Y) to ϕ^+_{AB} when $X = Y$, ϕ^-_{AB} when $X \neq Y \neq e$, and to $\pi_A \otimes |e\rangle\langle e|_B$ when $Y = e$ otherwise. As before, we make a specific choice of PPT* state in (18), but this time not a product state across channel uses. Instead, consider the classical distribution $P_X^{\times n} \times Q_{Y^n}$ given in [10, Eq. 168]. The Q_{Y^n} distribution has the property that any two y^n with the same number of erasure symbols e have the same probability, i.e. there is no dependence on the number of 0s versus 1s. The aforementioned map takes the distribution to a quantum state which is diagonal in the standard bases $\{|0\rangle, |1\rangle\}$ for A and $\{|0\rangle, |1\rangle, |e\rangle\}$ for B, and is therefore a PPT state. This can be seen as follows. Consider a fixed position j in a given a pair (x^n, y^n). If $y_j = e$, the state of the jth pair of systems AB is manifestly diagonal in the standard basis. On the other hand, if $y_j \neq e$, then the state is mapped to either ϕ^+_{AB} or ϕ^-_{AB} depending on the value of x_j. But the sequence in which y_j takes the other value has identical probability, meaning the two Bell states occur with equal probability, making the AB state diagonal. Since we may map $\omega_{AB}^{\otimes n}$ and $\sigma_{A^nB^n}$ to the associated classical distributions and back, the following converse holds for the qubit erasure channel,

$$\hat{R}^{\mathrm{cpp}}(n;\varepsilon) \leq \frac{1}{n} D_H^{\varepsilon}\left(P_{XY}^{\times n} \middle\| P_X^{\times n} \times Q_{Y^n}\right). \tag{35}$$

By design in the choice of σ_{AB}, this is precisely the bound for the BEC reported in [10, Thm. 38], as discussed in more detail in [13]. (This also corresponds to using Corollary 2 with $\mathcal{M}$ the channel which ignores its input and prepares σ_{B^n} at the output.)

Next, we construct an explicit coding scheme, involving classical post-processing including communication from the receiver to the sender, which matches the outer bound exactly. The strategy of the coding scheme is to generate maximally entangled qubit states using the quantum channels and then use the successfully transmitted (i.e. not erased) maximally entangled qubit states to distill a an entangled state of local dimension $|M|$, as required. Note that the number $|M|$ is fixed at the

outset of the code, i.e. the entanglement transmission scheme must deliver a maximally entangled state with local dimension $|M|$, possibly at the expense of low fidelity, rather than outputting a variable number of certifiably high fidelity entangled pairs.

The encoder prepares n maximally entangled qubit states $|\phi\rangle$ and sends one half of each over the channel. The other halves, together with the untouched system M', are stored in the memory register Q. The decoder now works as follows. The receiver determines which qubits have not been erased and informs the sender of their locations. Let L be the random variable indicating the total number of erasures and note that L follows a binomial distribution with parameters n and β. Let us also fix $k = \lceil \log |M| \rceil$ and consider the following two cases:

1. If $L = l \leq n - k$ the decoder can extract a maximally entangled state with unit fidelity. To do so, it selects k perfectly transmitted entangled qubits at the sender and receiver. Let us assume (without loss of generality) that these are in a state $|\phi^+\rangle^{\otimes k} = \frac{1}{\sqrt{2^k}} \sum_{i=1}^{2^k} |ii\rangle$.

   The receiver then prepares a maximally entangled state of local dimension $|M|$ by measuring the k qubits with the projective measure
$$\left\{ \frac{1}{\binom{2^k-1}{|M|-1}} \sum_{i \in \mathcal{S}} |i\rangle\langle i| : \mathcal{S} \subseteq \left[2^k\right] \wedge |\mathcal{S}| = |M| \right\} . \tag{36}$$
   The outcome, a subset $\mathcal{S}$ of cardinality $|M|$, is transmitted to the sender so that both sender and receiver now share a maximally entangled state on the subspace determined by $\mathcal{S}$.

2. On the other hand, if $L = l > n - k$ sender and receiver simply select the successfully transmitted qubits and embed them in a space of local dimension $|M|$. The fidelity with the target state $|\phi\rangle = \frac{1}{\sqrt{|M|}} \sum_{i=1}^{|M|} |ii\rangle$ is given by
$$F\big(|\phi^+\rangle\langle\phi^+|^{\otimes(n-l)}, \phi\big) = \frac{1}{|M|} \sum_{i,j=1}^{|M|} \langle i| \Big(|\phi^+\rangle\langle\phi^+|^{\otimes(n-l)} \Big) |j\rangle = \frac{2^{n-l}}{|M|} . \tag{37}$$

To complete the decoding operation, the sender and receiver perform quantum teleportation to teleport M' to the receiver, using the maximally entangled state prepared above as a resource. The fidelity of the state prepared above with the target state $\phi_{MM'}$ is then just the expected fidelity over L, which evaluates to
$$F = \sum_{l=0}^{n-k} \binom{n}{l} \beta^l (1-\beta)^{n-l} + \sum_{l=n-k+1}^{n} \binom{n}{l} \beta^l (1-\beta)^{n-l} \frac{2^{n-l}}{|M|} \tag{38}$$
$$= 1 - \sum_{l=n-k+1}^{n} \binom{n}{l} \beta^l (1-\beta)^{n-l} \left(1 - \frac{2^{n-l}}{|M|} \right) . \tag{39}$$

This is exactly the expression reported in the aforementioned outer bound in [10, Thm. 38], meaning the inner bound coincides with the outer bound when we allow classical post-processing and communication from the receiver to the sender.

Qubit Depolarizing Channel

The qubit depolarizing channel is covariant since it is a qubit Pauli channel. Using the Bell states $\phi^+_{AB} = \phi_{AA'}$, $\phi^-_{AB} = (1_A \otimes Z_{A'})\phi_{AA'}(1_A \otimes Z_{A'})$, $\psi^+_{AB} = (1_A \otimes X_{A'})\phi_{AA'}(1_A \otimes X_{A'})$, and

$\psi^-_{AB} = (1_A \otimes Y_{A'})\phi_{AA'}(1_A \otimes Y_{A'})$, we immediately find

$$\omega_{AB} := (\mathcal{I}_A \otimes \mathcal{D}_\alpha)(\phi_{AA'}) = (1-\alpha)\phi^+_{AB} + \frac{\alpha}{3}\left(\phi^-_{AB} + \psi^+_{AB} + \psi^-_{AB}\right) . \tag{40}$$

Now choosing $\sigma_{AB} = \frac{1}{2}\phi^+_{AB} + \frac{1}{6}(\phi^-_{AB} + \psi^+_{AB} + \psi^-_{AB})$ in (18) gives the outer bound

$$\hat{R}_{\mathcal{D}_\alpha}(n;\varepsilon) \leq \hat{R}^{\mathrm{cpp}}_{\mathcal{D}_\alpha}(n;\varepsilon) \leq \frac{1}{n} D^\varepsilon_H\left(\omega^{\otimes n}_{AB} \| \sigma^{\otimes n}_{AB}\right) . \tag{41}$$

As in the case of the qubit dephasing channel, we can convert the hypothesis test between ω_{AB} and σ_{AB} into a test between classical distributions, in fact precisely those distributions which were used in the dephasing example. This follows by considering the map which generates ϕ^+_{AB} when $X = Y$ and otherwise randomly generates one of the other Bell states when $X \neq Y$. Therefore, we obtain the same outer bound for the qubit depolarization channel as for the qubit dephasing channel.

Supplementary Note 4: Semidefinite Optimization

Here we describe how to formulate the outer bound from Theorem 4 in the main text as a semidefinite optimization program that satisfies strong duality.

A semidefinite program (SDP) is simply an optimization of a linear function of a matrix or operator over a feasible set of inputs defined by positive semidefinite constraints. We give only the bare essentials here, for more detail see [14, 15]. The maximization form of an SDP is defined by a Hermiticity-preserving superoperator $\mathcal{E}_{A\to B}$ taking $\mathcal{L}(A)$ to $\mathcal{L}(B)$, a constraint operator $C \in \mathcal{L}(B)$, and an operator $K \in \mathcal{L}(A)$ which defines the objective function. Here $\mathcal{L}(A)$ denotes the set of linear operators on A. The SDP is the following optimization, which we will also refer to as the primal form,

$$\begin{aligned} \alpha = \text{supremum} \quad & \mathrm{tr}[KX] \\ \text{subject to} \quad & \mathcal{E}(X) \leq C \\ & X \geq 0 . \end{aligned} \tag{42}$$

When the feasible set is empty, i.e. no X satisfy the constraints, we set $\alpha = -\infty$. The dual form arises as the optimal upper bound to the primal form, and takes the form

$$\begin{aligned} \beta = \text{infimum} \quad & \mathrm{tr}[CY] \\ \text{subject to} \quad & \mathcal{E}^\dagger(Y) \geq K \\ & Y \geq 0 . \end{aligned} \tag{43}$$

Again, when the set of feasible Y is empty, $\beta = \infty$. Weak duality is the statement that $\alpha \leq \beta$, that indeed the dual form gives upper bounds to the primal (or that the primal lower bounds the dual). Strong duality is the statement that the optimal upper bound equals the value of the primal problem, $\alpha = \beta$. This state of affairs often holds in problems of interest, and can be established by either of the following Slater conditions. In the first, called strict primal feasibility, strong duality holds if β is finite and there exists an $X > 0$ such that $\mathcal{E}(X) < C$. Contrariwise, under strict dual feasibility strong duality holds when α is finite and there exists a $Y > 0$ such that $\mathcal{E}^\dagger(Y) > K$. For strongly dual SDPs we also have the so-called complementary slackness conditions $\mathcal{E}^\dagger(Y)X = KX$ and $\mathcal{E}(X)Y = CY$ that relate the primal and dual optimizers.

Proposition 7. *With the notation from Corollary 2, the outer bound $f(\mathcal{N},\varepsilon)$ can be written as*

$$\begin{aligned} f(\mathcal{N},\varepsilon) \;=\; \text{minimum}\;\; & \operatorname{tr}[\xi_A] \\ \text{subject to}\;\; & \xi_A,\Gamma_{AB},\Lambda_{AB},\rho_A \geq 0 \\ & \xi_A\otimes 1_B \geq \Lambda_{AB} + \mathcal{T}_A(\Gamma_{AB}) \\ & \Lambda_{AB} \leq \rho_A \otimes 1_B \\ & |A|\operatorname{tr}[\Lambda_{AB}\,\mathcal{N}_{A'\to B}(\phi_{AA'})] \geq 1-\varepsilon \\ & \operatorname{tr}[\rho_A] = 1 \end{aligned} \tag{44}$$

or, equivalently,

$$\begin{aligned} f(\mathcal{N},\varepsilon) \;=\; \text{maximum}\;\; & \mu(1-\varepsilon)-\nu \\ \text{subject to}\;\; & \mu N_{AB} \leq M_{AB} + R_{AB} \\ & \operatorname{tr}_B[R_{AB}] \leq n 1_A \\ & M_{AB} \in \mathrm{PPT}(A\!:\!B) \\ & \operatorname{tr}_B(M_{AB}) \leq 1_A \\ & \mu,\nu,R_{AB} \geq 0\,. \end{aligned} \tag{45}$$

Here $PPT(A\!:\!B)$ denotes the subset of $\mathcal{S}(AB)$ consisting of positive operators with positive partial transpose.

Proof. The proof is straightforward: we simply use the dual of the inner optimization in (11) to obtain the minimization problem (44). Then we use Slater's condition to show that strong duality holds and obtain (45).

Consider the function

$$f_0(O_{AB}) := \max_{\mathcal{M}_{A\to B}\in \mathrm{PPT}} \operatorname{tr}[O_{AB}M_{AB}]\,, \tag{46}$$

over the domain $0 \leq O_{AB} \leq 1_{AB}$, and observe that f_0 is a semidefinite program. In particular, it is a primal problem as we have defined it, with $X = M_{AB}$, $K = O_{AB}$, $C = (0, 1_A)$, and $\mathcal{E}(X) = (-\mathcal{T}_A(X), \operatorname{tr}_B[X])$. Choosing for the dual variables $Y = (\Gamma_{AB}, \xi_A)$, the dual of f_0 is

$$\begin{aligned} \tilde{f}_0(O_{AB}) := \;\text{minimum}\;\; & \operatorname{tr}[\xi_A] \\ \text{subject to}\;\; & \xi_A\otimes 1_B \geq O_{AB} + \mathcal{T}_A(\Gamma_{AB}) \\ & \Gamma_{AB},\xi_A \geq 0\,. \end{aligned} \tag{47}$$

Combining this with the outer optimization in (11) gives the minimization program (44). The equality statement is precisely strong duality of the primal and dual forms of the inner optimization. By Slater's condition, strong duality holds if f_0 is finite and there exists a strictly feasible set of dual variables. Observe that $f_0(O_{AB}) \leq |A|$, since for the optimal M_{AB} we have $f_0(O_{AB}) = \operatorname{tr}[M_{AB}O_{AB}] \leq \operatorname{tr}[M_{AB}] \leq \operatorname{tr}_A[1_A] = |A|$. Here we have used the upper bounds $O_{AB} \leq 1_{AB}$ and $\operatorname{tr}_B[M_{AB}] \leq 1_A$. Thus, the first condition is fulfilled. Meanwhile, $\Gamma_{AB} = 1_{AB}$ and $\xi_A = 3\cdot 1_A$ are a strictly feasible pair. Thus, $\tilde{f}_0 = f_0$ over the domain of interest.

To construct the maximization program, we simply dualize the minimization program. In particular, $f(\mathcal{N},\varepsilon)$ is a dual-form semidefinite program in the variable $Y = (\phi_A, \Lambda_{AB}, \Gamma_{AB}, \xi_A)$ with $C = (0,0,0,1_A)$, $K = (1-\varepsilon, -1, 0, 0)$, and

$$\mathcal{E}^*(Y) = \Big(\operatorname{tr}[N_{AB}\Lambda_{AB}], -\operatorname{tr}[\phi_A], \mathcal{T}_A(\phi_A)\otimes 1_B - \Lambda_{AB}, \xi_A\otimes 1_B - \Lambda_{AB} - \mathcal{T}_A(\Gamma_{AB})\Big)\,. \tag{48}$$

Choosing primal variables $X = (m, n, R_{AB}, M_{AB})$ leads to the maximization in (45). Equality again follows from Slater's condition: f is finite (in particular the bound on f_0 used above), while a feasible choice of dual variables is given by $M_{AB} = R_{AB} = \frac{1}{2|B|}1_{AB}$, $n = 1$, and $m = \frac{1}{2|A||B|}$. The choice of m ensures the first constraint holds strictly, since any Choi operator of a trace-preserving map satisfies $\|N_{AB}\|_\infty = |A|$ (largest singular value). $\square$

No discussion of strong duality of semidefinite programs is complete until the complementary slackness conditions have been formulated. Often, these give considerable insight into the form and properties of the optimizing variables. First observe that

$$\mathcal{E}(X) = \Big(-n1_A + \mathrm{tr}_B[\mathcal{T}_A(R_{AB})],\, mN_{AB} - M_{AB} - R_{AB},\, -\mathcal{T}_A(M_{AB}),\, \mathrm{tr}_B[M_{AB}] \Big) . \tag{49}$$

Then the conditions are easy to read off from the form of C and K. They are

$$\mathrm{tr}[\phi_A] = 1 \tag{50}$$

$$\mathrm{tr}[\Lambda_{AB} N_{AB}] = 1 - \varepsilon \tag{51}$$

$$\mathcal{T}_A(\phi_A) R_{AB} = \Lambda_{AB} R_{AB} \tag{52}$$

$$\xi_A M_{AB} = (\Lambda_{AB} + \mathcal{T}_A(\Gamma_{AB}))\, M_{AB} \tag{53}$$

$$n\phi_A = \mathrm{tr}_B[\mathcal{T}_A(R_{AB})]\phi_A \tag{54}$$

$$\mathcal{T}_A(M_{AB})\Gamma_{AB} = 0 \tag{55}$$

$$\mathrm{tr}_B[M_{AB}]\xi_A = \xi_A \tag{56}$$

$$mN_{AB}\Lambda_{AB} = (M_{AB} + R_{AB})\Lambda_{AB} \, . \tag{57}$$

Supplementary References

[1] N. Datta, M. Tomamichel, and M. M. Wilde. Second-Order Coding Rates for Entanglement-Assisted Communication. 2014. arXiv: 1405.1797.

[2] E. M. Rains. Bound on Distillable Entanglement. *Physical Review A*, 60(1):179–184, 1999. DOI: 10.1103/PhysRevA.60.179.

[3] E. Rains. A Semidefinite Program for Distillable Entanglement. *IEEE Transactions on Information Theory*, 47(7):2921–2933, 2001. DOI: 10.1109/18.959270.

[4] P. Horodecki, M. Horodecki, and R. Horodecki. Binding entanglement channels. *Journal of Modern Optics*, 47(2):347, 2000. DOI: 10.1080/09500340008244047.

[5] M. Tomamichel, M. M. Wilde, and A. Winter. Strong Converse Rates for Quantum Communication. 2014. arXiv: 1406.2946.

[6] K. Li. Second-Order Asymptotics for Quantum Hypothesis Testing. *Annals of Statistics*, 42(1):171–189, 2014. DOI: 10.1214/13-AOS1185.

[7] M. Tomamichel and M. Hayashi. A Hierarchy of Information Quantities for Finite Block Length Analysis of Quantum Tasks. *IEEE Transactions on Information Theory*, 59(11):7693–7710, 2013. DOI: 10.1109/TIT.2013.2276628.

[8] C. Morgan and A. Winter. Pretty Strong Converse for the Quantum Capacity of Degradable Channels. *IEEE Transactions on Information Theory*, 60(1):317–333, 2014. DOI: 10.1109/TIT.2013.2288971.

[9] M. Hayashi and M. Tomamichel. Correlation Detection and an Operational Interpretation of the Renyi Mutual Information. 2014. arXiv: 1408.6894.

[10] Y. Polyanskiy, H. V. Poor, and S. Verdú. Channel Coding Rate in the Finite Blocklength Regime. *IEEE Transactions on Information Theory*, 56(5):2307–2359, 2010. DOI: 10.1109/TIT.2010.2043769.

[11] R. G. Gallager. *Information Theory and Reliable Communication*. Wiley, New York, 1968.

[12] G. Poltyrev. Bounds on the decoding error probability of binary linear codes via their spectra. *IEEE Transactions on Information Theory*, 40(4):1284–1292, 1994. DOI: 10.1109/18.335935.

[13] Y. Polyanskiy. Saddle Point in the Minimax Converse for Channel Coding. *IEEE Transactions on Information Theory*, 59(5):2576–2595, 2013. DOI: 10.1109/TIT.2012.2236382.

[14] S. Boyd and L. Vandenberghe. *Convex Optimization*. Cambridge University Press, 2004.

[15] J. Watrous. Theory of Quantum Information, Lecture Notes, 2011. Available online: https://cs.uwaterloo.ca/{~}watrous/quant-info/.

[16] D. Leung and W. Matthews. On the Power of PPT-Preserving and Non-Signalling Codes. 2014. arXiv: 1406.7142.
